# Solitonic solutions and stability analysis of Benjamin Bona Mahony Burger equation using two versatile techniques

**DOI:** 10.1038/s41598-024-60732-0

**Published:** 2024-06-12

**Authors:** Ejaz Hussain, Syed Asif Ali Shah, Abdul Bariq, Zhao Li, Muhammad Riaz Ahmad, Adham E. Ragab, Emad A. Az-Zo’bi

**Affiliations:** 1 Department of Mathematics, University of the Punjab, Quaid-e-Azam Campus, Lahore, 54590 Pakistan; 2 Department of Mathematics and Statistics, The University of Lahore, 1-km Defence Road, Lahore, 54000 Pakistan; 3 Department of Mathematics, Leghman University, 2701 Mehtarlam City, Laghman Afghanistan; 4 College of Computer Science, Chengdu University, 610106 Chengdu, People’s Republic of China; 5 Center for High Energy Physics, University of the Punjab, Quaid-e-Azam Campus, Lahore, 54590 Pakistan; 6 Department of Industrial Engineering, College of Engineering King Saud University, P.O. Box 800, 11421 Riyadh, Saudi Arabia; 7 Department of Mathematics, Mutah University, Mutah, Al Karak Jordan

**Keywords:** BBMB equation, New Kudryashov method, Extended $\exp (-\Psi (\chi ))$ expansion method, Stability analysis, Exact solitonic solutions, Mathematics and computing, Optics and photonics

## Abstract

This study aims to explore the precise resolution of the nonlinear Benjamin Bona Mahony Burgers (BBMB) equation, which finds application in a variety of nonlinear scientific disciplines including fluid dynamics, shock generation, wave transmission, and soliton theory. Within this paper, we employ two versatile methodologies, specifically the extended $\exp (-\Psi (\chi ))$ expansion technique and the novel Kudryashov method, to identify the exact soliton solutions of the nonlinear BBMB equation. The solutions we discovered involve trigonometric functions, hyperbolic functions, and rational functions. The uniqueness of this research lies in uncovering the bright soliton, kink wave solution, and periodic wave solution, and conducting stability analysis. Furthermore, the solutions’ graphical characteristics were explored through the utilization of the mathematical software Maple 2022 (https://maplesoft.com/downloads/selectplatform.aspx?hash=61ab59890f2313b2241fde3423fd975e). The system’s physical interpretation is defined through various types of graphs, including contour graphs, 3*D*-surface graphs, and line graphs, which use appropriate parameter values. These recommended techniques hold significant importance and are applicable in diverse nonlinear evolutionary equations found in the field of nonlinear sciences for illustrating nonlinear physical models.

## Introduction

The Benjamin–Bona–Mahony–Burger (BBMB) equation is a well-known nonlinear partial differential equation. It has attracted a significant amount of attention from both mathematicians and physicists due to the unique mathematical properties it possesses as well as the rich physical implications it carries. This equation was initially developed by Benjamin, Bona, Mahony, and Burger.

The use of nonlinear partial differential equations (NLPDEs) to explain a wide variety of models in mathematics and science is common knowledge. Hence, assessing recent solitary wave solutions to these equations is worthwhile. The BBMB equation is widely acknowledged as a useful mathematical model for the investigation of a wide variety of physical phenomena, such as fluid dynamics, shock production, wave propagation, and soliton theory. This recognition has led to the equation’s widespread use. It is a valuable instrument for researching wave phenomena and their interactions because it describes the evolution of a one-dimensional function that possesses dispersive and dissipative qualities.

This analysis of the BBMB equation is meant to be all-inclusive, embracing its theoretical underpinnings, mathematical properties, and important achievements gained to date. The purpose of this research paper is to present such an investigation. We hope to shed light on the fundamental behavior of the equation, uncover unanticipated phenomena, and provide pathways for further research by examining the most recent breakthroughs in analytical and numerical techniques.

Hence, several techniques for solving nonlinear partial differential equations have been developed over the past half-century by a wide range of scientists. such as the simple equation approach^1–4^, $(\frac{G'}{G})$-expansion method^5–7^, modified $(\frac{G'}{G})$ expansion method^8,9^, extended Jacobi elliptic function method^10,11^, $\exp (-\Psi (\chi ))$-expansion method^3,12,13^, extended $\exp (-\Psi (\chi ))$-expansion method^14^, Ricati-Bernoulli Sub-ODE method^15,16^, extended $\tanh$-function method^17–21^ and many more^22–32^.

The use of computational methods to achieve desirable outcomes in computational mathematics and symbolic programming played a significant role in the development (using tools like Matlab, Maple, Mathematica, etc.). Using computational tools, several researchers can propose new theories, domains, and solutions. Nonlinear wave propagation and nonlinear optics are unique subjects. Due to the increasing popularity of lasers and the concomitant growth of fiber technologies for use in communication and data transmission, many researchers have concentrated on these topics. The BBMB equation will be solved exactly and solitary wave solutions will be determined, using the new Kudryashov method and extended $\exp (-\Psi (\chi ))$-expansion method in this work.

Exploring the exponential technique, stability, and the new Kudryashov method is intriguing. However, the BBMB equation has not yet been analyzed using these approaches. The primary objective of this paper is to apply stability analysis and the new Kudryashov approach to the Benjamin–Bona–Mahony–Burger equation. Peregrine^33^ presented the nonlinear BBMB equation, which describes lengthy waves on the surface of the water in a channel with a small amplitude. This is the form of the BBMB equation:

1 $$\begin{aligned} \mathcal {U}_{t}+\mathcal {U}_{x}+\mathcal {U}\mathcal {U}_{x}-m \mathcal {U}_{xx}-\mathcal {U}_{xxt}=0, \end{aligned}$$

where the subscripts represent the partial derivatives of position *x* and time *t*, u(x, t) is a real-valued function that is believed to be a dissipative term and *m* is any real number. The nonlinear BBMB equation is addressed, and several updated precise wave solutions are presented.

The structure of this paper is organized as follows: Sect. "Mathematical analysis" provides a detailed formulation of the New Kudryashov Method and extended$\exp (-\Psi (\chi ))$ expansion technique is described and derivation of the BBMB equation, elucidating its physical interpretation and mathematical basis. Sections "Solution by new Kudryashov method", [Sec Sec7]" presents a survey of notable solutions and exact solutions obtained for the BBMB equation, highlighting their significance and implications. Section "Graphical explanation" graphical interpretation is described. Section "Stability analysis" concludes the stability analysis of the paper and the final paragraph provides a synopsis of the conclusion by summarizing the main findings and discussing potential directions for future research.

## Mathematical analysis

Assume that the general nonlinear partial differential equations (NLPDEs) have the form as follows:

2 $$\begin{aligned} M(\mathcal {U},\mathcal {U}_{x}, \mathcal {U}_{t},\mathcal {U}_{xt},\mathcal {U}_{tt}, \mathcal {U}_{xxx}.~.~.~)=0, \end{aligned}$$

where $\mathcal {U}=\Psi (x,t)$ is unknown function.

By traveling wave transformation, we can convert NLPDEs into ordinary differential equations which have the form as follows:

3 $$\begin{aligned} \mathcal {U}(x,t)=\Psi (\chi ),~\chi =ax+bt, \end{aligned}$$

Put Eq. (3) into Eq. (2) as a result, we obtain the ODE of the form

4 $$\begin{aligned} M(\Psi ,~\Psi ',~\Psi '',~\Psi ''',~...)=0 \end{aligned}$$

where *a* is any arbitrary constant and *b* is speed of soliton. Assuming that Eq. (1) admits the traveling wave transformation to discover the precise solution of Eq. (1) using wave transformation,

5 $$\begin{aligned} \mathcal {U}(x,t)=\Psi (\chi ),~\chi =ax+bt. \end{aligned}$$

The following equation is derived by inserting Eq. (5) into Eq. (1) and we get nonlinear (ODE) of the form,

6 $$\begin{aligned} b\Psi '+a\Psi '+a\Psi \Psi '-ma^{2}\Psi ''-a^{2}b\Psi '''=0. \end{aligned}$$

Integrate the above expression and put the constant of integration equal to zero then we get.

7 $$\begin{aligned} b\Psi +a\Psi +a\frac{1}{2}\Psi ^{2}-ma^{2}\Psi '-a^{2}b\Psi ''=0. \end{aligned}$$

Here *a*, and *m* are arbitrary constants and *b* is our soliton wave speed.

### New Kudryashov method

Assume that the Eq. (7) has the solution of the form as follows^34,35^:

8 $$\begin{aligned} \Psi (\chi )=\sum _{j=1}^{N}c_{j}Q^{j}({\chi }), \end{aligned}$$

where coefficients $c_{j}(j=0,1,2...N)$ are constants to be determined such that $c_{j}\ne 0$,and $Q^{j}({\chi })=\frac{1}{r A^{\Theta \chi }+s A^{-\Theta \chi }}$ is solution of nonlinear ODE.

9 $$\begin{aligned} Q'({\chi })^{2}= (\Theta (\ln A)Q{\chi })^{2}(1-4 r s Q({\chi })^{2}). \end{aligned}$$

Where the constant $r,s, \Theta$ and *A* are non-zero with $A >0$ and $A \ne 1$. First, we find the positive value of *N* which can be determined by the homogeneous balance principle by comparing the linear term with the highest order and nonlinear tern with the highest degree and then inserting the value of *N* in Eq. (8) and Eq. (8) put in Eq. (7). Since $Q({\chi })\ne 0$, and we equating the different power of $Q({\chi })$ to zero, we obtain the system of equation and solve the system of equation $c_{j}$ and *b* are determined.

### The extended $\exp (-\Psi (\chi ))$-expansion method

The general solution of Eq. (4) in $\exp (-\Psi (\chi ))$-expansion method^36^ is given by

10 $$\begin{aligned} \Psi (\chi )=\sum _{i=0}^{N}B_{i}(\exp (-\Psi (\chi )))^{i}, \end{aligned}$$

where $\Psi (\chi )$ satisfies the following ODE and $B_{i}\ne {0}$ are constants to be calculated.

11 $$\begin{aligned} \Psi '(\chi )=\exp (-\Psi (\chi ))+\rho \exp (\Psi (\chi ))+\eta . \end{aligned}$$

As arbitrary constants in Eq. (11), $\eta$ and $\rho$. Following are the considerations for the answers to Eq. (11).

- If $\eta ^{2}-4\rho >0$ and $\rho \ne 0$, then
  12 $$\begin{aligned} \Psi (\chi )=\ln \left( \frac{-\sqrt{\eta ^{2}-4\rho } \tanh (\frac{\sqrt{\eta ^2-4\rho }}{2}(\chi +h))-\eta }{2\rho }\right) . \end{aligned}$$
- If $\eta ^{2}-{4}\rho >0$ and $\rho =0$ and $\eta \ne 0$, then
  13 $$\begin{aligned} \Psi (\chi )=-\ln \left( \frac{\eta }{\cosh (\eta (\chi +h))+\sinh (\eta (\chi +h))-1}\right) . \end{aligned}$$
- If $\eta ^{2}-{4}\rho =0$, $\eta =0$ and $\rho =0$, then
  14 $$\begin{aligned} \Psi (\chi )=\ln (\chi +h). \end{aligned}$$
- If $\eta ^{2}-4\rho =0$, $\eta \ne 0$ and $\rho \ne 0$, then
  15 $$\begin{aligned} \Psi (\chi )=\ln \left( -\frac{2(\eta (\chi +h))+2}{\eta ^{2}(\chi +h)}\right) . \end{aligned}$$
- If $\eta ^{2}-4\rho <0$ and $\rho \ne 0$, then
  16 $$\begin{aligned} \Psi (\chi )=\ln \left( \frac{\sqrt{4\rho -\eta ^{2}}\tan {\left( \frac{\sqrt{4\rho -\eta ^{2}}}{2}(\chi +h)\right) }-\eta }{2\rho }\right) . \end{aligned}$$

### Ethics approval and consent to participate

The authors declare that there is no conflict with publication ethics.

## Solution by new Kudryashov method

Solution of Eq. (7) by New Kudryashov method Eq. (8), comparing the highest derivative term $\Psi ''$ with nonlinear term $\Psi ^{2}$, and we obtain value of $N=2$ and put in Eq. (8) we get:

17 $$\begin{aligned} \Psi (\chi )= & {} c_{0} + c_{1}Q(\chi )+ c_{2}Q^{2}(\chi ). \end{aligned}$$

Substituting Eq. (17) in Eq. (7) and equating the coefficient of different power of $Q(\chi )$ equal to zero, then we obtain the following system of equations.

18 $$\begin{aligned} \left. \ \begin{array}{lll} Q^{0}&{}:&{} -m a A + a c_0 + b c_0 + \frac{a c_0^2}{2}=0,\\ Q^{1}&{}:&{}-\ln (A)^2 \Theta ^2 a^2 b c_{1} + a c_{0} c_{1} + a c_{1} + b c_{1}=0,\\ Q^{2}&{}:&{} -4c_{2}\ln (A)^2a^2b\Theta ^2 + ac_{0}c_{2} + ac_{2} + c_{2}b + \frac{ac_{1}^2}{2}=0,\\ Q^{3}&{}:&{} 8\ln (A)^2\Theta ^2a^2brc_{1}s + ac_{1}c_{2}=0,\\ Q^{4}&{}:&{}24\ln (A)^2\Theta ^2a^2brsc_{2} + \frac{ac_{2}^2}{2}=0.\\ \end{array}\right\} \end{aligned}$$

By solving the system of algebraic equations simultaneously, we can determine solutions for the given set of equations.

**Set 1.**

$$\begin{aligned} a= & {} \frac{\sqrt{-\frac{-2A m - c_{0}^2}{8A m - 4c_{0}^2 - 8c_{0}}}}{\ln (A) \Theta },~~ b = \frac{\sqrt{-\frac{-2A m - c_{0}^2}{8A m - 4c_{0}^2 - 8c_{0}}} (2A m - c_{0}^2 - 2c_{0})}{2 \ln (A) \Theta c_{0}},\\ c_{0}= & {} c_{0},~~ c_{1} = 0,~~ c_{2} = -\frac{6 s r (2A m + c_{0}^2)}{c_{0}}. \end{aligned}$$

Inserting the Set 1 in Eq. (17) we recover the exact solution as follows:

$$\begin{aligned} \Psi _{1}(x,t)= c_{0} - \frac{6 s r (2A m + c_{0}^2)}{c_{0} (r A^{\Theta \chi } + s A^{-\Theta \chi })^2}. \end{aligned}$$

## Solution by the extended $\exp (-\Psi (\chi ))$-expansion method

In order to discover the precise solutions of Eq. (1), the general solution for $N=2$ is expressed as,

$$\begin{aligned} \Psi (\chi ) = B_{0}+B_{1}\exp (-\Psi (\chi ))+B_{2}\exp (-\Psi (\chi ))^{2}. \end{aligned}$$

By equating the different power of $\exp (-\Psi (\chi ))$ to zero and we get following set algebraic equation,

$$\begin{aligned} \left. \begin{array}{lll} e^{-\Psi (\chi )^{0}}: a^{2}bB_{1}\eta ^{2}\rho +6a^{2}bB_{2}\eta \rho ^{2}-ma^{2}B_{1}\eta \rho +2a^{2}bB_{1}\rho ^{2} -2ma^{2}B_{2}\rho ^{2}\\ -a\rho B_{1}B_{0}-a\rho B_{1}-b\rho B_{1}=0,\\ e^{-\Psi (\chi )^{1}}: a^{2}\eta ^{3}bB_{1}+14a^{2}\eta ^{2}b\rho B_{2}-a^{2}\eta ^{2}mB_{1}+8a^{2}\eta b\rho B_{1}-6a^{2}\eta \rho mB_{2}\\ +16a^{2}b\rho ^{2}B_{2}-2a^{2}\rho mB_{1}-a\eta B_{0}B_{1}-2aB_{0}B_{2}\rho -a\rho B_{1}^{2}-a\eta B_{1}\\ -2a\rho B_{2}-\eta b B_{1}-2b\rho B_{2}=0,\\ e^{-\Psi (\chi )^{2}}: 8a^{2}\eta ^{3}bB_{2}+7a^{2}\eta ^{2}bB_{1}-4a^{2}\eta ^{2}mB_{2}+52a^{2}\eta b\rho B_{2}-3a^{2}\eta mB_{1}\\ +8a^{2}b\rho B_{1}-8a^{2}\rho mB_{2}-2a\eta B_{0}B_{2}-a\eta B_{1}^{2}-3a\rho B_{1}B_{2}-2a\eta B_{2}\\ -aB_{0}B_{1}-2\eta bB_{2}-aB_{1}-bB_{1}=0,\\ e^{-\Psi (\chi )^{3}}: 38a^{2}\eta ^{2}bB_{2}+12a^{2}\eta bB_{1}-10a^{2}\eta mB_{2}+40a^{2}b\rho B_{2}\\ -2a^{2}mB_{1}-3a\eta B_{1}B_{2}-2a\rho B_{2}^{2}-2aB_{0}B_{2}-aB_{1}^{2}-2aB_{2}-2bB_{2}=0,\\ e^{-\Psi (\chi )^{4}}: 54a^{2}\eta bB_{2}+6a^{2}bB_{1}-6a^{2}mB_{2}-2a\eta B_{2}^{2}-3aB_{1}B_{2}=0,\\ e^{-\Psi (\chi )^{5}}: 24a^{2}B_{2}-2aB_{2}^{2}=0.\\ \end{array}\right\} \end{aligned}$$

By simultaneously solving the recovered system of algebraic equations, we can find solutions to the following set of equations:

**Set 1.**

$$\begin{aligned} a= & \, a,~ b=\sqrt{\frac{1}{25\eta ^{2}-100\rho }}.m,~ \\ B_{0}= & \, \frac{1}{25}\sqrt{\frac{1}{25\eta ^{2}-100\rho }}.\frac{1}{a}\left[ \frac{25a^{2}\eta ^{2}m}{25\eta ^{2}-100\rho }+\frac{200a^{2}\rho m}{25\eta ^{2}-100\rho }-30\sqrt{\frac{1}{25\eta ^{2}-100\rho }}a^{2}\eta m\right. \\{} & {} \left. -\frac{25m}{25\eta ^{2}-100\rho }-a^{2}m-25\sqrt{\frac{1}{25\eta ^{2}-100\rho }}a \right] ,\\ B_{1}= & \, 12\sqrt{\frac{1}{25\eta ^{2}-100\rho }}ma\eta -\frac{12}{5}am,\\ B_{2}= & \, 12\sqrt{\frac{1}{25\eta ^{2}-100\rho }}ma \end{aligned}$$

**Family 1.**

- When $\eta ^{2}-4\rho >0$ and $\rho \ne 0$, then
  19 $$\begin{aligned} \Psi _{2}(x,t)= & {} \frac{1}{25}\frac{1}{\sqrt{\frac{1}{25\eta ^{2}-100\rho }}a}\left[ \frac{25a^{2}\eta ^{2}m}{25\eta ^{2}-100\rho }+\frac{200 a^{2}\rho m}{25\eta ^{2}-100\rho }-30\sqrt{\frac{1}{25\eta ^{2}-100\rho }}a^{2}\eta m\right. \nonumber \\{} & {} \left. -\frac{25m}{25\eta ^{2}-100\rho }-a^{2}m-25\sqrt{\frac{1}{25\eta ^{2}-100\rho }}a\right] +\frac{2\left( 12\sqrt{\frac{1}{25\eta ^{2}-100\rho }}am\eta -\frac{12am}{5}\right) \rho }{-\sqrt{\eta ^{2}-4\rho }\tanh \left( \frac{1}{2}\sqrt{\eta ^{2}-4\rho }(\chi +h)\right) -\eta }\nonumber \\{} & {} +\frac{{48\sqrt{\frac{1}{25\eta ^{2}-100\rho }}ma\rho ^{2}}}{\left( -\sqrt{\eta ^{2}-4\rho }\tanh (\frac{1}{2}\sqrt{\eta ^{2}-4\rho }(\chi +h))-\eta \right) ^{2}}, \end{aligned}$$
- When $\eta ^{2}-4\rho >0$, $\eta \ne 0$ and $\rho =0$, then
  20 $$\begin{aligned} \Psi _{3}(x,t)= & \, \frac{1}{25}\frac{1}{\sqrt{\frac{1}{25\eta ^{2}-100\rho }}a}\left[ \frac{25a^{2}\eta ^{2}m}{25\eta ^{2}-100\rho }+\frac{200a^{2}\rho m}{25\eta ^{2}-100\rho }-30\sqrt{\frac{1}{25\eta ^{2}-100\rho }}a^{2}\eta m\right. \nonumber \\{} & {} \left. -\frac{25m}{25\eta ^{2}-100\rho }-a^{2}m-25\sqrt{\frac{1}{25\eta ^{2}-100\rho }}a \right] + \frac{12\left( \sqrt{\frac{1}{25\eta ^{2}-100\rho }}ma\eta -\frac{12}{5}ma\right) \eta }{\cosh (\eta (\chi +h))+\sinh (\eta (\chi +h))-1}\nonumber \\{} & {} +\frac{12\sqrt{\frac{1}{25\eta ^{2}-100\rho }}ma\eta ^{2}}{\left( \cosh (\eta (\chi +h))+\sinh (\eta (\chi +h))-1\right) ^{2}}, \end{aligned}$$
- When $\eta ^{2}-4\rho =0$, $\rho \ne 0$, and $\eta \ne 0$, then
  21 $$\begin{aligned} \Psi _{4}(x,t)= & \, \frac{1}{25}\left( \frac{25a^2\alpha ^2m}{25\alpha ^2-100\beta }+\frac{200a^2\beta m}{25\alpha ^2-100\beta }-30\sqrt{\frac{1}{25\alpha ^2-100\beta }}a^2\alpha m-\frac{25m}{25\alpha ^2-100\beta }\right. \nonumber \\{} & {} \left. -a^2m-25\sqrt{\frac{1}{25\alpha ^2-100\beta }}a\right) \frac{\sqrt{\frac{1}{25\alpha ^2-100\beta }}a}{a}\nonumber \\{} & {} -\frac{1}{2}\left( 12\sqrt{\frac{1}{25\alpha ^2-100\beta }}ma\alpha -\frac{12}{5}am\right) \frac{a(\chi +h)^2}{a(\chi +h)+2}\nonumber \\{} & {} +3\sqrt{\frac{1}{25\alpha ^2-100\beta }}ma\frac{a(\chi +h)^4}{(a(\chi +h)+2)^2}. \end{aligned}$$
- When $\eta ^{2}-4\rho =0$, $\rho =0$, and $\eta =0$, then
  22 $$\begin{aligned} \Psi _{5}(x,t)= & {} \frac{1}{25}\left( \frac{25a^2\alpha ^2m}{25\alpha ^2-100\beta }+\frac{200a^2\beta m}{25\alpha ^2-100\beta }-30\sqrt{\frac{1}{25\alpha ^2-100\beta }}a^2\alpha m-\frac{25m}{25\alpha ^2-100\beta }\right. \nonumber \\{} & {} \left. -a^2m-25\sqrt{\frac{1}{25\alpha ^2-100\beta }}a\right) \left( \frac{1}{\sqrt{\frac{1}{25\alpha ^2-100\beta }}a}\right) \nonumber \\{} & {} +\frac{12\sqrt{\frac{1}{25\alpha ^2-100\beta }}ma\alpha -\frac{12}{5}am}{\chi +h}+\frac{12\sqrt{\frac{1}{25\alpha ^2-100\beta }}ma}{(\chi +h)^2}. \end{aligned}$$
- When $\eta ^{2}-4\rho <0$ and $\rho \ne 0$, then
  23 $$\begin{aligned} \Psi _{6}(x,t)= & {} \frac{1}{25}\frac{1}{\sqrt{\frac{1}{25\eta ^{2}-100\rho }}a}\left[ \frac{25a^{2}\eta ^{2}m}{25\eta ^{2}-100\rho }+\frac{200a^{2}\rho m}{25\eta ^{2}-100\rho }-30\sqrt{\frac{1}{25\eta ^{2}-100\rho }}a^{2}\eta m\right. \nonumber \\{} & {} \left. -\frac{25m}{25\eta ^{2}-100\rho }-a^{2}m-25\sqrt{\frac{1}{25\eta ^{2}-100\rho }}a \right] + \frac{2\left( 12\sqrt{\frac{1}{25\eta ^{2}-100\rho }}ma\eta -\frac{12}{5}ma\right) \rho }{\sqrt{-\eta ^{2}+4\rho }tan\left[ \frac{1}{2}\sqrt{-\eta ^{2}+4\rho }(\chi +h)\right] -\eta } \nonumber \\{} & {} +\frac{48\sqrt{\frac{1}{25\eta ^{2}-100\rho }}ma\rho ^{2}}{\left( \sqrt{-\eta ^{2}+4\rho }tan\left[ \frac{1}{2}\sqrt{-\eta ^{2}+4\rho }(\chi +h)\right] -\eta \right) ^{2}}. \end{aligned}$$

## Graphical explanation

This portion of the study explores the graphical properties of the created solutions. To build representations of the computed solutions in 3*D*, 2*D*, and a contour graph for various values of the arbitrary parameters, precise values are provided. The illustrations in portion (a) display a 3*D* surface plot, where the solutions 2*D*-contour behavior is represented in section (b), and (c) depicts the line graph for different values of *t*.

**Figure 1:**
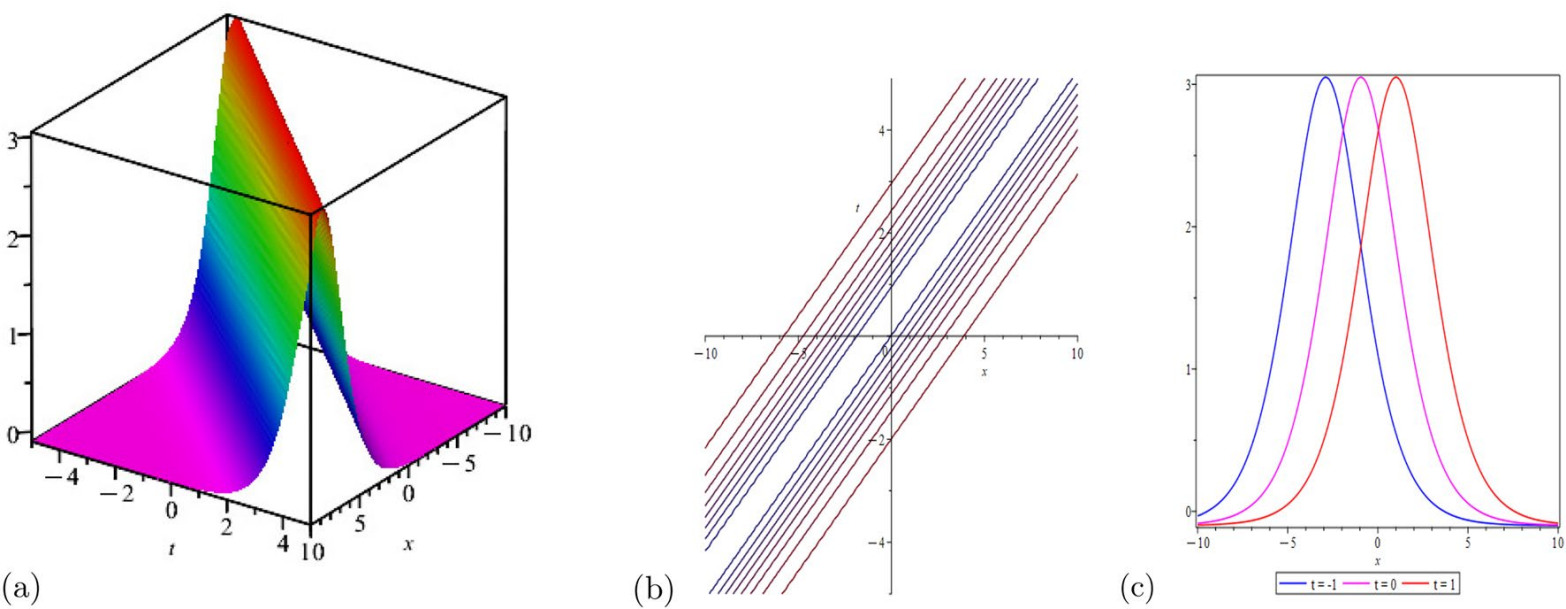
Shows the behavior of bright soliton solution with, $\Psi _{1}(x,t)$: $r=1$, $c_{0}=$-0.1 $s=0.5$, $A=2$, $m=0.05$, $\Theta =1$, for line graph$t=1$.

Figure 1 shows the behavior of bright soliton solution with, $\Psi _{1}(x,t)$: $r=1$, $c_{0}=-0.1$ $s=0.5$, $A=2$, $m=0.05$, $\Theta =1$, for line graph$t=1$

**Figure 2:**
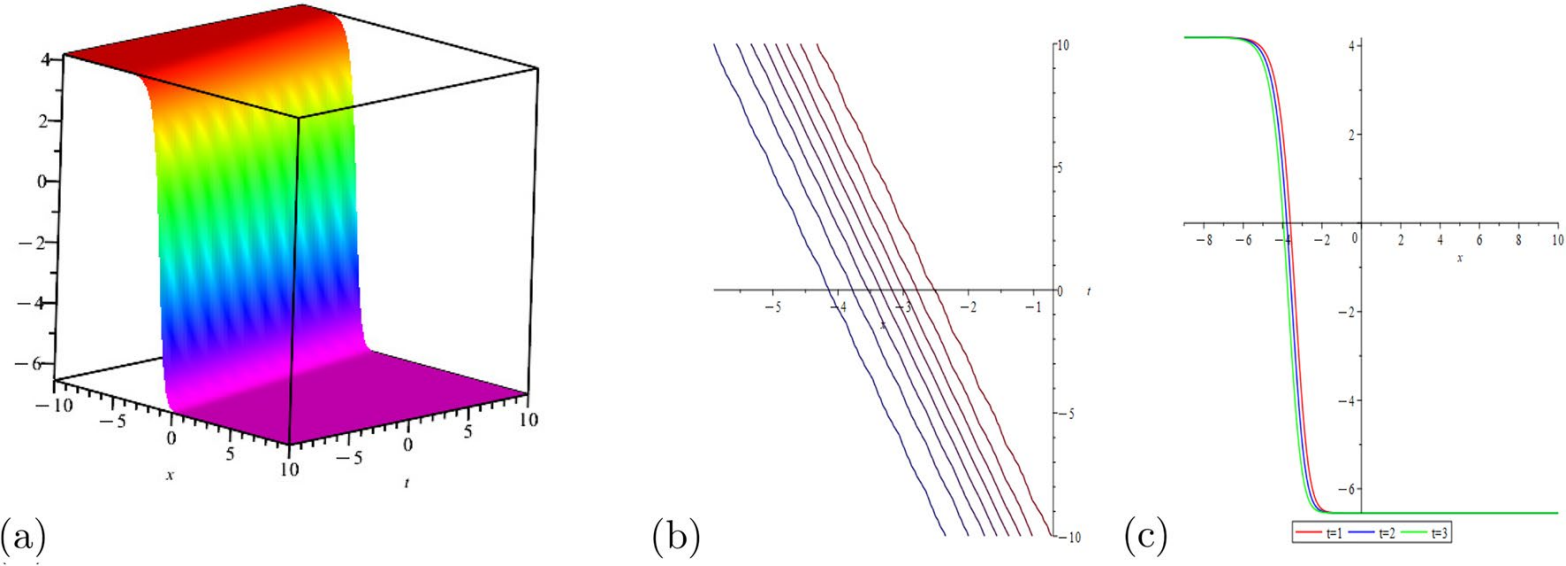
Shows the behavior of kink soliton solution with,$\Psi _{2}(x,t)$: $a=1$, $\eta =3$ $\rho =1$, $h=2$, $m=2$, $\chi =ax+bt$, for line graph$t=1$.

**Figure 3:**
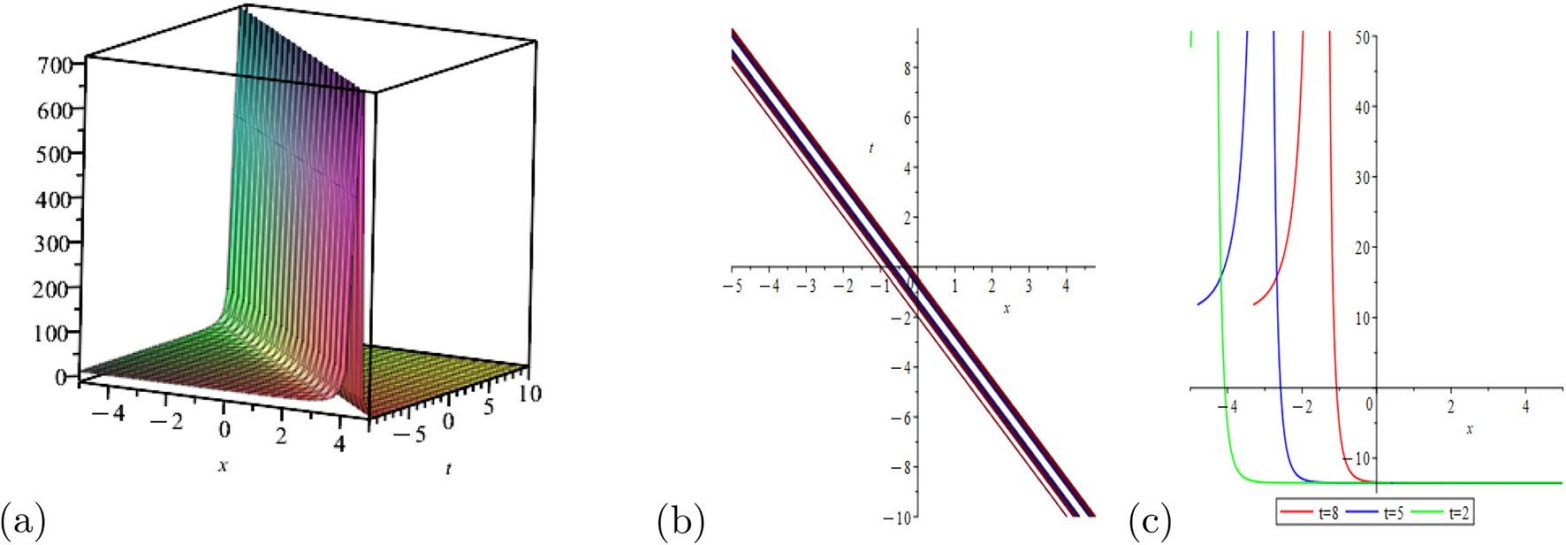
Shows the behavior of bright soliton solution, $\Psi _{3}(x,t)$: $a=2$, $\eta =1$, $\rho =0$, $h=1$, $m=5$, $\chi =ax+bt$, for line graph$t=1$.

**Figure 4:**
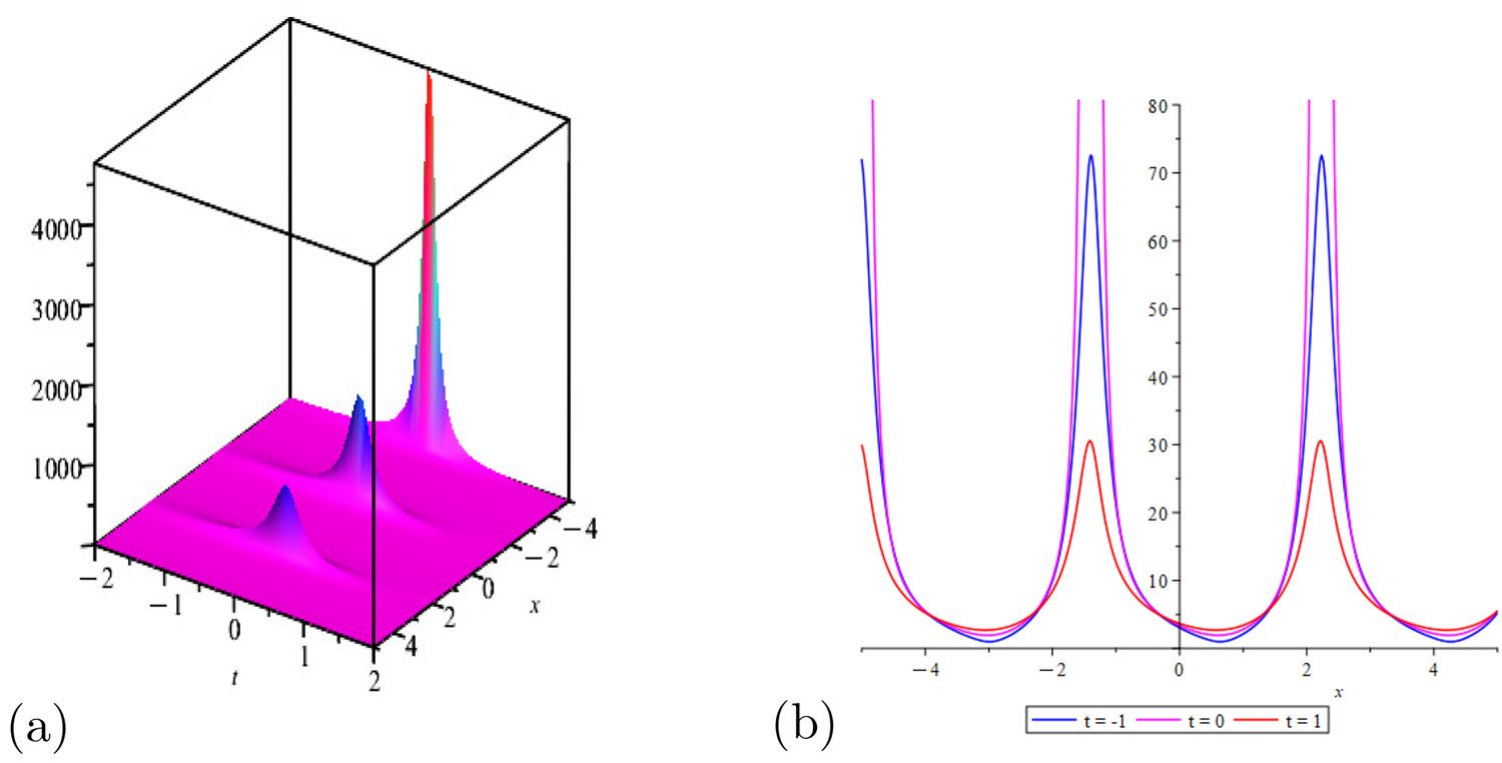
Shows the behavior of periodic wave solution,$\Psi _{6}(x,t)$: $a=1$, $\eta =1$, $\rho =1$, $m=2$, $\chi =ax+bt$, for line graph$t=1$.

Figure 2 shows the behavior of kink soliton solution with,$\Psi _{2}(x,t)$: $a=1$, $\eta =3$ $\rho =1$, $h=2$, $m=2$, $\chi =ax+bt$, for line graph$t=1$. Figure 3 shows the behavior of bright soliton solution, $\Psi _{3}(x,t)$: $a=2$, $\eta =1$, $\rho =0$, $h=1$, $m=5$, $\chi =ax+bt$, for line graph$t=1$. Figure 4 shows the behavior of periodic wave solution,$\Psi _{6}(x,t)$: $a=1$, $\eta =1$, $\rho =1$, $m=2$, $\chi =ax+bt$, for line graph$t=1$. This research additionally discusses the graphical behavior of solutions generated using the extended $\exp (-\Psi (\chi ))$ expansion method and the new Kudryashov method.

## Stability analysis

In this part, we will use linear stability analysis to examine the stability of the provided Eq. (1). We shall examine the model’s altered solution to ascertain its stability characteristics^37^.

24 $$\begin{aligned} \Psi (x,t)= S+\lambda W(x,t). \end{aligned}$$

It can be easily shown that the equation (1) possesses a stable state solution for any constant value of *S*. When we substitute equation (24) into equation (1), the resulting expression is as follows:

25 $$\begin{aligned} \lambda W_{t}+\lambda W_{x}+S\lambda W_{x}+\lambda ^{2}WW_{x}-m\lambda W_{xx}-\lambda W_{xxt}=0. \end{aligned}$$

By use of the linearization method, the following result has been attained.

26 $$\begin{aligned} \lambda W_{t}+\lambda W_{x}+S\lambda W_{x}-m\lambda W_{xx}-\lambda W_{xxt}=0 . \end{aligned}$$

Eq. (33) has the solution of the form:

27 $$\begin{aligned} W(x,t)=e^{\iota (kx-\sigma t)}, \end{aligned}$$

where *k* and $\sigma$ are wave number and dispersion relation respectively, putting the Eq. (27) in Eq. (26) then we obtained the following result:

28 $$\begin{aligned} \sigma (k)=\frac{m k^{2}+\iota k +\iota S k}{\iota (1+k^{2})}. \end{aligned}$$

We examine the propagation properties given in Eq. (28) in this section. A key factor in deciding whether the solution will expand or decay over time is the sign of $\sigma (k)$. The dispersion relation shown in the equation is stable due to several processes, including self-phase modulation, group velocity dispersion, and stimulated Raman scattering (28).

The solutions hold steady even in the presence of disturbances when the wave numbers *k* and *S* are non-negative (actual values). Exponential distortion and unstable steady-state solutions arise when the wave numbers become infinite, specifically when $k=\iota$ (an imaginary value). As a result, demonstrating stability becomes relatively straightforward when $k=\iota$”.

## Conclusion

The principal objective of the investigation was to examine the Benjamin–Bona–Mahony–Burgers equation, a nonlinear partial differential equation incorporating an additional dispersion term. This particular equation is widely utilized to model fluid dynamics, wave propagation, and soliton theory. A variety of accurate solutions to the BBMB equation were obtained in this study by employing the novel Kudryashov method and the technique of $\exp (-\Psi (\chi ))$-expansion. These methodologies were utilized to generate multiple precise solutions to the extant issue. As a result, a diverse array of innovative exact traveling wave solutions were unveiled, including bright solitons, kink wave solutions, and periodic wave solutions. Bright solitons are representations of localized energy peaks that can propagate without experiencing any dissipation. Kink waves, on the other hand, are propagating waves that are characterized by variations in amplitude, which can either increase or decrease as they transition from one asymptotic state to another. The methodology is straightforward, reliable, and pragmatic, rendering it essential to be employed across a broad spectrum of other Nonlinear Partial Differential Equations (NLPDEs). Furthermore, it can be utilized for the resolution of various other partial differential equations within the realm of physics. The utilization of this approach across a diverse array of additional NLPDEs is of paramount importance. The graphical behavior of the solutions is scrutinized and elucidated through the manipulation of the arbitrary constants’ values. The methodologies employed could potentially provide novel insights and understanding of these mathematical expressions, potentially leading to new findings across various scientific fields. The findings of the research are anticipated to have a significant impact on the fields of optical fibers and the telecommunications industry.

## Data Availability

The data that support the findings of this study are available from the corresponding author upon reasonable request.
